# Supplementary material for: Spin-orbit driven superconducting proximity effects in Pt/Nb thin films
Source: Nat Commun. 2023 Aug 21;14:5081. doi: 10.1038/s41467-023-40757-1 (PMC10442328; doi:10.1038/s41467-023-40757-1)
Supplement: Supplementary file 1 — Supplementary Information [file 41467_2023_40757_MOESM1_ESM.pdf]

Supplementary Material for "Spin-orbit driven superconducting proximity effects in Pt/Nb thin films" by M. G. Flokstra et al.

This supplementary materials contains additional information about 1) the theoretical framework used to describe our data and 2) the experimental techniques used: LEM, STS, TEM and transport. While not essential to the understanding or conclusions of the manuscript, specialist readers may find the additional information useful and informative.

A. Quasiclassical Theory

To compare our experimental results with theory we make use of the quasiclassical framework in the dirty limit (coherence length much longer than the mean free path), where the Green functions obey the Usadel equation [1]. To simulate our scanning tunneling spectroscopy data we require the electronic density of states, which follows directly from the obtained Green functions. For our muon experiments, where we measure local flux, we apply the linear response theory to calculate the screening current in response to a small externally applied field.

We use realistic material parameters for our sample layouts, which where possible we determine directly from independent measurements. The parameters required to describe the interfaces between two materials are tuned such that the predicted superconducting transition temperature matches the experimentally observed value. Furthermore, we initially omit spin-orbit interaction as well as additional pair-breaking mechanisms. We find this describes our system well as long as the Pt layers are very thin ($\leq$ 10 nm), but for our sample using $\sim$ 50 nm thick Pt layers a clear discrepancy between the flux expulsion observed in our muon experiments and the prediction from theory emerges. For this system with thick Pt, we use a detailed theoretical approach including spin-orbit interactions which is able to explain the muon data. Below follows first the theory modeling omitting spin-orbit interactions, and its application to the STS data (which requires the self-consistent solution for $\Delta(x)$) and the LEM data (which requires the solution for $B(x)$). The theory including spin-orbit is tailored for sample SM3 and is detailed under the section: Triplet correlations induced by impurity spin-orbit interaction.

We take the x-axis normal to the metallic layers and assume translational invariance in the y,z plane. The Usadel equation for s-wave superconductivity then takes the form $i\hbar D\partial_x\left(\check{g}\partial_x\check{g}\right) = \left[\check{H},\check{g}\right]$ with $\check{g}$ the 4×4 matrix Green function in the Nambu $\otimes$ spin-space, $\hbar$ the reduced Planck constant and D the diffusion constant (see e.g. [2]). The Hamiltonian can be described by $\check{H} = i\hbar\omega_n(\tau_3\otimes\sigma_0)+\check{\Delta}$ with ω_n the Matsubara frequencies defined by $\hbar\omega_n = \pi k_B T(2n+1)$ with k_B the Boltzmann constant, n integer and the maximum allowed frequency given by the Debye frequency. Furthermore, σ_i and τ_i are the Pauli matrices of the spin space and Nambu space respectively. The matrix Green function and $\check{\Delta}$ only have non-zero elements on their main and anti-diagonals with diag($\check{g}$) = $\left(G_{\uparrow\uparrow}, G_{\downarrow\downarrow}, \overline{G}_{\uparrow\uparrow}, \overline{G}_{\downarrow\downarrow}\right)$, anti-diag($\check{g}$) = $\left(F_{\uparrow\downarrow}, F_{\downarrow\uparrow}, \overline{F}_{\uparrow\downarrow}, \overline{F}_{\downarrow\uparrow}\right)$ and anti-diag($\check{\Delta}$) = $(-\Delta, \Delta, -\Delta^*, \Delta^*)$, where G and F are the quasiclassical normal and anomalous Green functions respectively, both being functions of (x, ω_n), $\Delta(x)$ is the order parameter and where up/down arrows indicate spin-up/spin-down. The matrix Green function satisfies the normalization condition $\check{g}^2 = \check{1}$ and the order parameter must be solved self-consistently satisfying the gap equation:

$$i\Delta(x) = \frac{\pi k_B T}{\ln\left(\frac{T}{T_{c0}}\right) + \sum_n\left(\frac{1}{|2n+1|}\right)} \sum_{\omega_n} F_{\uparrow\downarrow}(x,\omega_n) \tag{1}$$

with T_{c0} the bulk critical temperature. We use the interface boundary conditions as formulated by Nazarov[3], which for the interface between two materials with labels l, r for the layer on the left and right side of the interface respectively can be written as: $\sigma_l\check{g}_l\partial_x\check{g}_l = \sigma_r\check{g}_r\partial_x\check{g}_r$ and $\sigma_l\check{g}_l\partial_x\check{g}_l = \frac{2}{R_b}\frac{\check{g}_l\check{g}_r - \check{g}_r\check{g}_l}{4+\Gamma(\check{g}_l\check{g}_r+\check{g}_r\check{g}_l-2)}$, with σ_i the conductivity of layer i, $0 \leq \Gamma \leq 1$ the interface transparency and R_b the interface resistance times the interface area.

When the Green functions are known one can calculate the response of the superconductor to a (small) external field. Within the linear response theory, the shielding current density $j_y(x)$ in response to the vector potential $A_y(x)$ is written as[4]:

$$j_y = -\frac{N_0 e^2 D}{\hbar} A_y \pi k_B T \sum_{\omega_n} \text{Real}\left(F_{\uparrow\downarrow}\overline{F}_{\downarrow\uparrow} + F_{\downarrow\uparrow}\overline{F}_{\uparrow\downarrow}\right) \tag{2}$$

with N_0 the normal state density of states near the Fermi energy. The vector potential is defined by $\mathbf{B} = \nabla \times \mathbf{A}$ and using Maxwell's equation $\nabla \times \mathbf{B} = \mu_0 \mathbf{j}$, the current density must thus satisfy $\mu_0 \mathbf{j} = \nabla \times (\nabla \times \mathbf{A})$. Using both expressions for the current density the vector potential (and thus the magnetic flux $\mathbf{B}$) can be solved.
To obtain the electronic density of states, one needs to determine the real-time, energy (ϵ) dependent Green functions rather than the imaginary-time ones. Using the Keldysh technique, the matrix Green function and Hamiltonian (used in the Usadel equation) are now written as:

$$\check{g} = \begin{pmatrix} \check{g}^R & \check{g}^K \\ 0 & \check{g}^A \end{pmatrix}, \check{H} = \begin{pmatrix} \check{H} & 0 \\ 0 & \check{H} \end{pmatrix} \tag{3}$$

Here, $\check{g}^R$ and $\check{g}^A$ are the retarded and advanced components describing equilibrium properties and $\check{g}^K$ is the Keldysh component which describes the non-equilibrium properties. Each component is a 4×4 matrix Green function in the Nambu $\otimes$ spin-space and a function of (x, ϵ). As long as the system is in equilibrium it is fully determined by the retarded component and it is thus sufficient to calculate the retarded equation, which effectively replaces $\check{g}(x, \omega)$ by $\check{g}^R(x, \epsilon)$ and $i\hbar\omega_n$ by ϵ. Once the retarded Green functions are obtained, the electronic density of states ($N(x, \epsilon)$) follows from taking the real part of the (retarded) normal Green function.

B. STS

The scanning tunneling microscopy/spectroscopy (STM/S) measurements were performed using a home-built, cryo-pumped low temperature STM machine operating at a base temperature below 2 K. The samples were placed in the entry-lock chamber of the machine and pumped at a vacuum pressure of 8×10^{-6} mbar overnight, before they were introduced to the STM image. The STM tip was made from PtIr wire, which before use was conditioned by field emission on a Au single crystal target. Bias voltage (V) was applied to the sample with the STM tip at virtual ground. Differential conductance (dI/dV) versus V spectroscopy data were recorded using the standard lock-in technique, with the frequency and amplitude of voltage modulation set at 413 Hz and 0.2 mV, respectively.
The differential conductance of the STS spectra are modeled as [5]:

$$\frac{dI}{dV} = G_0 \int N(\epsilon) \left(-\frac{\partial f(\epsilon + eV)}{\partial(eV)} \right) d\epsilon, \tag{4}$$

with G_0 the differential conductance at e$V >> \Delta$, N the (normalized) density of states (DOS) at the sample surface, f the quasiparitcle distribution function and V the applied voltage bias (with respect to the grounded sample) to the STM tip. For the N we use the results obtained from the quasiclassical theory and also make a comparison with the phenomenological Dynes theory [6] which approximates the DOS by:

$$N_{\mathrm{Dy}}(\epsilon, \Delta, \Gamma) = \mathrm{Real}\left(\frac{z}{\sqrt{z^2 - \Delta^2}} \right), \text{ with } z = |\epsilon| + i\Gamma \tag{5}$$

where Δ is a measure of the (induced) superconducting gap and Γ the effective pair-breaking strength.
Figure S1 shows in more detail the analysis presented in Fig. 1 of the manuscript and includes a direct comparison to fitting the data with the Dynes model. The latter predicts stronger smearing of the peak features than what is actually measured and gives less compelling fits compared to using a more realistic DOS obtained from quasiclassical simulations. Figure S2 shows $\Delta(T)$ obtained from both approaches, where for the quasiclassical approach (where Δ is solved as function of position) $\Delta(T)$ is shown both at the sample surface and at the Nb/Pt interface. Note that Δ as used in the quasiclassical theory itself is zero outside the superconductor and what is actually shown is the imaginary part of $\sum F_{\uparrow\downarrow}$, scaled such that for bulk Nb it gives the correct bulk value for the gap (Δ_0). The obtained full spatial dependence of Δ (normalized to Δ_0) for each temperature is shown in Fig. S3. Figure S4 shows the STM topographic image for the Au(5 nm)/Pt(2 nm)/Nb(50 nm) sample.

C. LEM

All low-energy muon-spin rotation (LE-μSR) measurements were performed on the μE4 beamline at the Paul Scherrer Institut [7]. The positive muon is an unstable spin-$\frac{1}{2}$ lepton of charge $+e$ with a lifetime $\tau_\mu = 2.197\mu$s and on decay emits a positron preferentially along its momentary spin direction. Upon implantation into a material a muon will rapidly thermalize, while maintaining its spin direction, after which its spin precesses around the local field making it a local magnetic probe. By monitoring the decay positrons of implanted, 100% spin polarized muons, information

about the precession frequency (and thus the local field) can be obtained. The implantation depth profile (or stopping profile) of the muon is energy dependent and can be calculated by a well-proven Monte-Carlo simulation[8, 9]. This allows the average probing depth to be tuned from about 10 to 100 nm below the surface. For a typical measurement several million counting events are collected at a rate of about 1 k/s and errors as small as 0.1 G can be achieved[10]. All our muon measurements were undertaken in transverse field geometry (applied field orthogonal to the muon spin direction) with the applied field direction in the plane of the sample. From the measurement data taken at a particular muon energy E (and thus a particular probing depth profile) one can determine the average flux $\langle B\rangle(E)$, which can also be presented as $\langle B\rangle(\langle x\rangle)$ with $\langle x\rangle$ the average probing depth of the muons at energy E. However, in cases where the shape of the flux profile is known, either analytically including free fit parameters or numerically, one can treat the measurement data imposing this $B(x)$ to find best fits to the data. To compare this to the conventional approach it is necessary to convolute $B(x)$ with the muon stopping profiles in order to estimate which $\langle B\rangle(E)$ the profile corresponds to.

An example measurement of a muon experiment is shown in Fig. S5 (including the corresponding muon stopping profiles) where $N_{\mathrm{L}}(t)$ and $N_{\mathrm{R}}(t)$ are the measured positron detector histograms of the left and right detector respectively and t is the time at which a positron is detected. The two detectors are placed on opposite sides of the sample and the implanted muons have their initial spin direction pointing towards the left detector, with the result that from all the muons that decay at $t = 0$ a bigger fraction hits the left detector compared to the right detector. As a function of time, the muon-spin precesses around the local field which results in the muon-spin direction alternating between the left and right detector. The main exponential decay of the measurement signal reflects the muon life-time and the superimposed damped oscillations contain the information about the local field and define the asymmetry $A(t)$ of the signal. The histograms can be fitted using $N_{\mathrm{L,R}}(t) = N^0_{\mathrm{L,R}}\,(1 \pm A(t))\exp(-t/\tau_\mu) + K_{\mathrm{L,R}}$ with N^0 the amplitude of the signal, K the time-independent background contribution and $A(t)$ the asymmetry of the signal which carries all the information concerning the field distributions. It can generally be modeled as $A(t) = \int dx A_0 p(x)\cos(\gamma_\mu B(x)t + \phi)\,G(t)$, with A_0 the (setup dependent) maximum asymmetry that can be measured, $p(x)$ the muon stopping profile, $\gamma_\mu = 851$ Mrad·s^{-1} the gyromagnetic ratio for the muon, $B(x)$ the local flux density, ϕ the starting angle of the muon-spin direction and $G(t)$ the depolarization function ($G \leq 1$), where the integral runs over the full width of the stopping profile

In Fig. S6 we present muon data obtained on a Pt(60nm)/Nb(50nm). The results are similar to those obtained on sample SM3 of the main manuscript, but due to the superconductor being much thinner (50 nm instead of 96 nm for SM3), the effect (e.g. difference between the measurement in the normal state compared to the superconducting state) is also much reduced and only just visible. In Fig. S7 we present muon data obtained on a Pt(94nm)/Nb(96nm), which compared to sample SM3 has a thicker Pt layer. Up to the highest muon energy used, where muons reach up to some 20-25 nm into the Nb layer (see Fig. S7), no signs of a net Meissner screening is observed. This apparent lack of Meissner screening is consistent with our experimental findings on SM3 and related theory modeling. Due to the Pt layer being thicker (94 nm compared to 56 nm for SM3), higher muon energies are required in order for them to reach the Pt/Nb interface, which also results in a more spread-out stopping profile.

D. Triplet correlations induced by impurity spin-orbit interaction

For sample SM3 we use a detailed quasi-classical simulation including spin-orbit interaction in order to find a realistic solution for the local magnetic flux density, and from that, using the muon stopping profiles, the average flux density that would be observed during a muon experiment.

For the theoretical explanation of the experimental results we make use of the quasiclassical framework with extrinsic spin-orbit coupling (SOC) in the dirty limit [11–13] (coherence length much longer than mean free path), where the Green functions obey the Usadel equation. It is known that both intrinsic and extrinsic SOC induce singlet-triplet conversion at hybrid interfaces of usual singlet superconductors with nonsuperconducting materials, even in the absence of the exchange field [11, 12, 14–19]. In the framework of the considered model triplets are generated by the Meissner currents in the presence of the impurity SOC, quantified by the spin Hall angle θ. The triplets are small and their amplitude relative to the singlet component is controlled by the parameter $p_s\xi\theta$, where p_s is the condensate momentum and ξ is the superconducting coherence length. This parameter is very small and our estimates give $p_s\xi \sim 0.05$ for the system under consideration (at the applied field $B = 300$ G and the Nb thickness 96 nm). Therefore, we can consider the triplet correction as a small perturbation. Then the general Usadel equation [11–13] can be simplified and up to the first order with respect to the parameter $p_s\xi\theta$ takes the form:

$$\left[\omega_n\hat{\tau}_z - i\check{\Delta}, \check{g}\right] + \hat{\partial}_k \check{J}_k = 0, \tag{6}$$

where $\check{g}$ is the quasiclassical Green's function, which is a 4×4 matrix in the direct product of particle-hole and spin spaces. We use Pauli matrices $\hat{\tau}_i$ for the particle-hole space and $\hat{\sigma}_i$ for the spin space. Eq. (6) is written in the

Matsubara representation and ω_n is the Matsubara frequency. $\check{\Delta} = |\Delta|\hat{\tau}_3 e^{i\tau_3\chi}\tau_1$, where $|\Delta|$ is the absolute value of the superconducting order parameter and χ is its phase, which in our case can be set equal to zero.

$$\hat{\partial}_k = \partial_k - \frac{ie}{c}\left[A_k\hat{\tau}_z, ...\right], \tag{7}$$

where A_k is the k-coordinate component of the vector potential of the magnetic field.

$$\check{J}_k = -D\Big(\check{g}\hat{\partial}_k\check{g} + \frac{\theta}{2}\varepsilon_{akj}\big\{\hat{\partial}_j\check{g}, \hat{\sigma}_a\big\}\Big), \tag{8}$$

where D is the diffusion constant and θ is the spin Hall angle of the material. The main generator of the triplets is the second term in the round brackets in Eq. (8). The applied magnetic field generates Meissner currents in the superconductor, which lead to the appearance of the triplets via this term. Please note that the sign of θ is changed with respect to Ref. [12]. In this case the parameter θ exactly coincides with the spin Hall angle, defined as in the limit of the nonsuperconducting system. Below we consider the process in detail.

If the magnetic field is applied along the z axis, the only nonzero component of the vector potential is A_y and nonzero $\hat{\partial}_y\check{g}$ appears accounting for the Meissner currents along the y direction. Then the second term in the round brackets in Eq. (8) results in nonzero spin component $\propto \sigma_z$ of the matrix current $\check{J}_x$. Further it serves as a generator of the opposite-spin triplets with $S_z = 0$, which are described by the anomalous Green's function $(f_s + f_t\sigma_z)$.

For the problem under consideration it is convenient to calculate the Green's function via the Θ-parametrization [20]. The matrix structure in the particle-hole space is written explicitly:

$$\check{g} = \begin{pmatrix} \cosh\Theta & \sinh\Theta \\ -\sinh\Theta & -\cosh\Theta \end{pmatrix} + \\ \delta\Theta \begin{pmatrix} \sinh\Theta & \cosh\Theta \\ -\cosh\Theta & -\sinh\Theta \end{pmatrix}\hat{\sigma}_z \tag{9}$$

The singlet contribution to the anomalous Green's function is contained in the first term of Eq. (9) and is represented by $\sinh\Theta$. The triplet contribution is contained in the second term and is represented by $\delta\Theta\cosh\Theta$. The both quantities depend on the coordinate x along the direction perpendicular to the S/N interface. $\delta\Theta \propto p_s\xi\theta$, as it is demonstrated below, and, therefore, small. Further we substitute the ansatz Eq. (9) into the Usadel equation Eq. (6) and up to the first order with respect to the parameter $p_s\xi$ obtain the following equations for $\Theta(x)$ and $\delta\Theta(x)$:

$$-D\partial_x^2\Theta + 2\big(i\Delta(x)\cosh\Theta + \omega_n\sinh\Theta\big) = 0, \tag{10}$$

$$-D\partial_x^2\delta\Theta + 2\delta\Theta\big(i\Delta(x)\sinh\Theta + \omega_n\cosh\Theta\big) + \\ \frac{2ie}{c}D\theta B\sinh\Theta = 0. \tag{11}$$

Eq. (11) contains the term depending on the spin Hall angle θ, which could be a generator of the triplet correlations. However this term is typically small for type-II superconductors being of the order of ξ/λ with respect to all the other terms. The main generator of the triplets appears via the boundary conditions at the S/N interface. The boundary conditions [12] represent the generalized Kupriyanov-Lukichev boundary conditions and take the form:

$$\check{J}_{x,N} = \frac{D_N}{2R_b\sigma_N}\left[\check{g}_S, \check{g}_N\right], \tag{12}$$

$$N_N\check{J}_{x,N} = N_S\check{J}_{x,S}, \tag{13}$$

where $\check{J}_{x,N(S)}$ and $\check{g}_{N(S)}$ are the values of the x-component of the matrix current Eq. (8) and the Green's function, respectively, at the normal $x = -0$ (superconducting $x = +0$) side of the interface. $N_{N(S)}$ is the normal state density of states at the Fermi level in the normal (superconducting) part of the hybrid structure, $\sigma_{N(S)}$ are the conductivities and R_b is the interface resistance per unit area. In terms of the Θ-parametrization Eqs.(12)-(13) take the form:

$$\partial_x\Theta_N = \frac{1}{R_b\sigma_N}\sinh(\Theta_S - \Theta_N) \tag{14}$$

$$\sigma_N\partial_x\Theta_N = \sigma_S\partial_x\Theta_S, \tag{15}$$

$$\partial_x\delta\Theta_N + ip_s\theta_N\sinh\Theta_N = \\ \frac{1}{R_b\sigma_N}(\delta\Theta_S - \delta\Theta_N)\cosh(\Theta_S - \Theta_N) \tag{16}$$

$$\sigma_N[\partial_x\delta\Theta_N + ip_s\theta_N\sinh\Theta_N] = \\ \sigma_S[\partial_x\delta\Theta_S + ip_s\theta_S\sinh\Theta_S]. \tag{17}$$

Here $p_s = -2(e/c)A_y$ is the condensate momentum due to the Meissner currents and $\theta_{N,S}$ are the spin Hall angles in the normal and superconducting parts of the structure. Eqs. (16) and (17), which represent boundary conditions for $\delta\Theta$, contain terms $\propto p_s\theta_{N,S}$, which are generators for the triplets.

The superconducting order parameter should be calculated self-consistently according to:

$$\Delta(x) = \pi i \lambda T \sum_{\omega_n} \sinh \Theta(x). \tag{18}$$

The spatial distribution and the amplitude of the triplet anomalous Green's function $f_t = \delta\Theta \cosh\Theta$ is presented in Fig. S9 for the Pt/Nb system for several Matsubara frequencies. As it should be for dirty samples, f_t is an example of odd-frequency s-wave superconducting correlations. The lengths of layers are chosen to fit the experimental sample SM3. The resistance of the Pt/Nb interface is $R_b = 2 \cdot 10^{-15}$ $\Omega\mathrm{m}^2$, the diffusion constants are $D_{Nb} = 3.6 \cdot 10^{-4}$ m^2/c and $D_{Pt} = 1.1 \cdot 10^{-3}$ m^2/c, the conductivities are $\sigma_{Nb} = [1.5 \cdot 10^{-7}\ \Omega\mathrm{m}]^{-1}$ and $\sigma_{Pt} = [1.1 \cdot 10^{-7}\ \Omega\mathrm{m}]^{-1}$. In agreement with the structure of Eqs. (16) and (17) the amplitude of the triplet component at the interface should be of the order of $f_t(x=0) \sim \theta p_s \xi f_s(x=0)$. In our case $f_s(\omega_1) \sim 1$ and $p_s\xi \sim 0.05$ at $B = 300$ G. Taking $\theta_{Pt} = 0.096$ (see below) we obtain that the numerical value of $f_{t,Pt}(\omega_1, x=0)$ is in reasonable agreement with these rough estimates.

In principle, the triplet component contributes to the measured magnetic field in two different ways: (i) it results in the spin splitting of the quasiparticle DOS caused by the spin-triplet pairs. Although the triplet pairs have zero spin projection onto the z-axis, they are inevitably accompanied by the appearance of the spin dependence of the normal Green's function, as it is suggested by Eq.(9). In its turn, the spin-dependent correction to the normal Green's function leads to the spin splitting of the DOS, giving rise to the magnetization; (ii) it leads to the paramagnetic contribution to the Meissner currents. The distribution of the magnetic field in the system can be found making use of the Maxwell's equation:

$$\mathrm{rot\ rot}\mathbf{A} = \frac{4\pi}{c}\big(\mathbf{j_s} + c\ \mathrm{rot}\mathbf{M}\big), \tag{19}$$

In the considered geometry Eq. (19) can be rewritten in the form:

$$-\frac{d^2A_y}{dx^2} = -4\pi\frac{dM_z}{dx} + \frac{4\pi}{c}QA_y. \tag{20}$$

The first term in the right-hand side of Eq. (20) accounts for the the magnetization created by the triplet pairs and the second one describes paramagnetic contribution to the Meissner currents. We consider the both effects and have to conclude that the second one is negligibly small. The paramagnetic contribution to j_s can be estimated in terms of the paramagnetic correction to the kernel $Q = Q_s(1+\delta Q)$, where Q_s is the singlet contribution to the Meissner response. The magnitude of $\delta Q \propto (p_s\xi)^2\theta^2$, in fact it is even smaller because of additional reducing factors coming from the exact solution. From the numerical calculation it is obtained that in the framework of the considered mechanism it cannot account for the observed paramagnetic response and even does not make a visible contribution to it. Therefore, we focus only on the magnetization, induced by the triplet pairs. It can be calculated as

$$M_z = \frac{2i\pi\mu_B N_F T}{1+F_0^a}\sum_{\omega} \sinh\Theta\delta\Theta, \tag{21}$$

where F_a is the Landau Fermi liquid parameter [21] and can be expressed via the experimentally accessible normal state susceptibility $\chi = 2\mu_B^2 N_F/(1+F_0^a)$. Then the magnetization takes the form

$$M_z = \frac{i\pi T\chi}{\mu_B}\sum_{\omega} \sinh\Theta\delta\Theta. \tag{22}$$

The direct measurements of the low temperature normal state susceptibility of a Pt(40nm) film grown on a Si substrate give the result $4\pi\chi = 0.104/(T+0.652)$ in Gaussian units (dimensionless).

The spatial profile of the corresponding paramagnetic field contribution $\delta B = 4\pi M_z$, calculated according to Eqs. (20) and (22), is presented in Fig.4(c) of the manuscript. To compare the calculated profile with the muon results it is necessary to convolute $\delta B(x)$ with the energy-dependent muon stopping profile. In order to compare $\delta B(x=0)$ at the interface with the experimental results presented in Fig.4(b) of the manuscript, we convolute $\delta B(x)$ with the muon stopping distribution corresponding to $19(54)keV(nm)$ (approximately at the interface). Fitting the result of the convolution, which is denoted by $\langle B_+\rangle$ to the experimental value we obtain $\theta_{Pt} = 0.096$. We also assume nonzero value of the impurity SOC in Nb $\theta_{Nb} = -0.2\theta_{Pt}$. Under this assumption we obtain small nonzero value of the triplet component in the depth of Nb, which is maintained by the Meissner currents, that is decays over the length

scale of the magnetic field penetration depth. It results in the nonzero negative magnetization deep in Nb in Fig.4(c) of the manuscript. However, the small spin Hall effect in Nb does not influence the result qualitatively.

In order to compare the temperature of $\delta B(T)$ at a given x with the experimental results presented in Fig.5(a)-(b) of the manuscript we convolute $B(x)$ with the muon stopping distribution corresponding to 19(54) keV(nm) (approximately at the interface) and 27(82) keV(nm) (in the depth of Nb). The result of the convolution of δB, which is denoted by $\langle B_+ \rangle$, is presented in Fig. S10 as a function of temperature. The green curve corresponds to the convolution of δB with the muon distribution 19(54) keV(nm) and the black curve is for the convolution with 27(82) keV(nm) distribution. It is seen that $\langle B_+ \rangle$ is strongly suppressed with increasing temperature. We see three reasons for this suppression. The strongest effect comes from the temperature suppression of the triplet correlations, the second reason is the temperature dependence of the normal state susceptibility χ. The third reason is associated to the fact that the decay length of δB in Pt, which is, roughly speaking, nothing but the normal state coherence length $\xi_N = \sqrt{D_{pt}/2\pi T}$ declines with increasing temperature. After the convolution procedure it leads to smaller $\langle B_+ \rangle$. Fig.5(c) of the manuscript represents the result of the convolution of the total magnetic field, which is a sum of the diamagnetic response of the singlet component and the paramagnetic contribution of the triplet component.

The paramagnetic contribution becomes smaller with shortening the Pt layer. It is connected to the fact that the triplets generated at the opposite interfaces of the Pt layer have opposite signs if the directions of p_s are the same at those interfaces. The same effect has been discussed in Ref. [11]. The paramagnetic contribution should become stronger in case of further increase of the Nb length. Roughly speaking, the effect is proportional to the Nb length unless it reaches 2 magnetic field penetrating depths of Nb. The point is that the triplet f_t is proportional to the value $p_s(x=0)$ at the Nb/Pt interface. For the structures under consideration the dependence $p_s(x)$ is nearly linear with the zero crossing point corresponding approximately to the middle of the Nb layer. Then $p_s(x=0) = (2e/c)BL/2$, where L is the Nb length.

E. Transport

Transport measurements were performed in a commercial 7 T helium flow cryostat using a He-3 insert (Cryogenic Mini Cryogen Free System). A standard four-point geometry was used to measure resistance (R) as function of temperature (T) and applied field (H). Figure S11 shows the results obtained on the Cu/Pt/Nb (SM2) sample. The $R(H)$ curves are measured both for decreasing and increasing field but no significant hysteresis was observed. From the field decreasing branches, at fixed temperature, and always starting from fields above the critical field to ensure normal state, the upper (perpendicular) critical fields are determined ($H_{\mathrm{c2,perp}}$). The obtained $H_{\mathrm{c2,perp}}(T)$ are fitted to $\mu_0 H_{\mathrm{c2,perp}}(T=0)(1-(T/T_c(H=0))^{\alpha})^{\beta}$ and the Ginzburg-Landau coherence length is determined from $\xi_{\mathrm{GL}} = \sqrt{\hbar/(2e\mu_0 H_{\mathrm{c2,perp}}(T=0))}$.

F. SEM/TEM

Samples for transmission electron microscopy (TEM) were prepared by a focused ion beam technique using a FEI Scios Dualbeam FIB. Sites of interest were identified by SEM imaging and protected from the damaging effects of the gallium ion beam by the electron-deposition of a carbon layer followed by the application of a thicker gallium ion-deposited platinum layer. Thin foils were then prepared by ion milling and transferred in-situ to a copper TEM grid for final thinning and ion-polishing. TEM and scanning transmission electron microscopy (STEM) were carried out using a FEI Titan Themis S/TEM operated at 200 kV equipped with a Super-X windowless x-ray energy dispersive spectrometer (EDS). Bruker Esprit software was used for analysis and quantification of EDS spectra. Figure S12 shows the EDS analysis of the Pt/Nb sample (SM3).

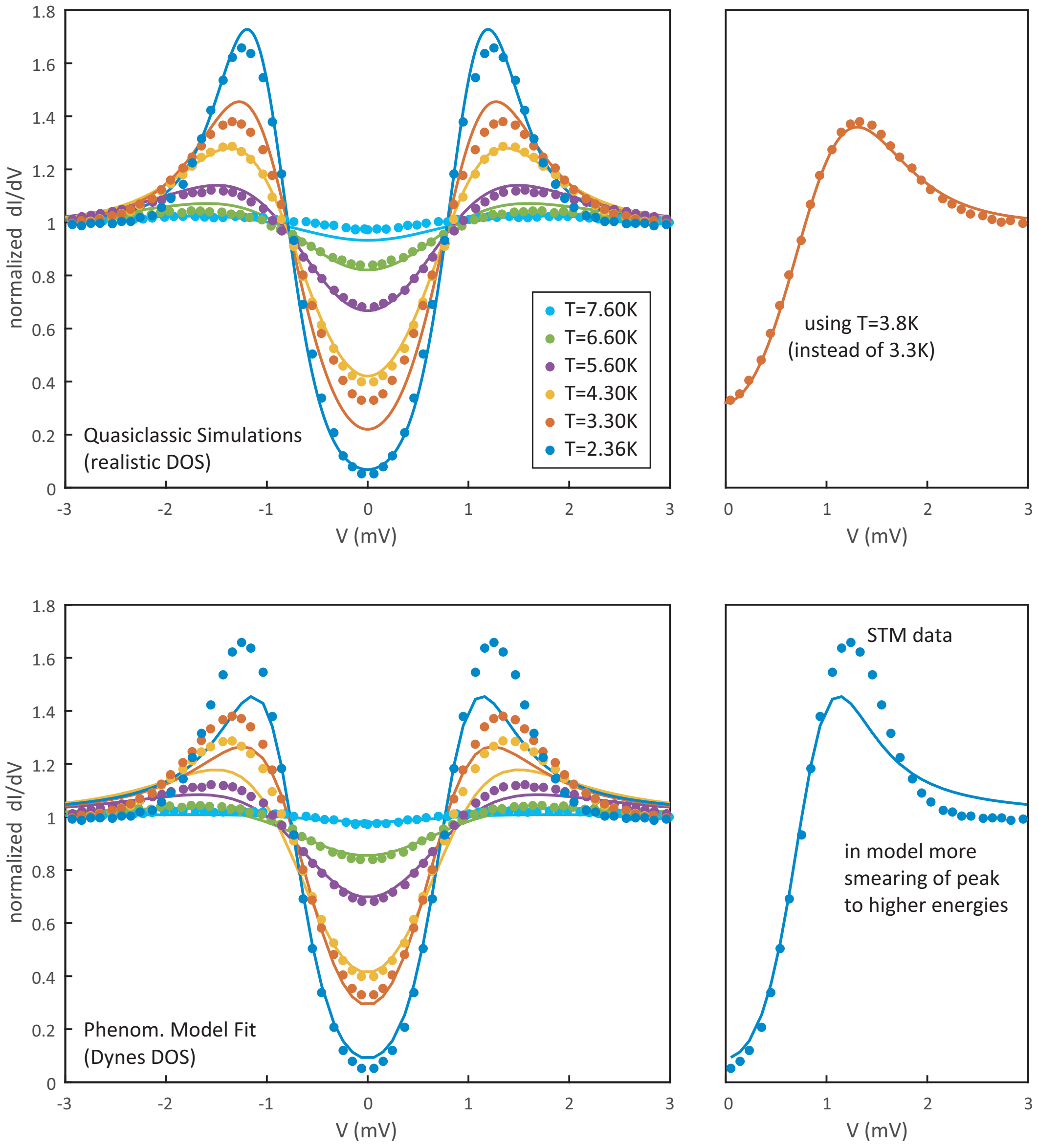

FIG. S1: STS data for sample ST2 (see manuscript, Fig.1) with a direct comparison between using the quasiclassical theory to calculate a realistic DOS and the phenomenological Dynes model for the DOS. The STS spectra taken from 2.36 K to 4.3 K have been symmetrized.

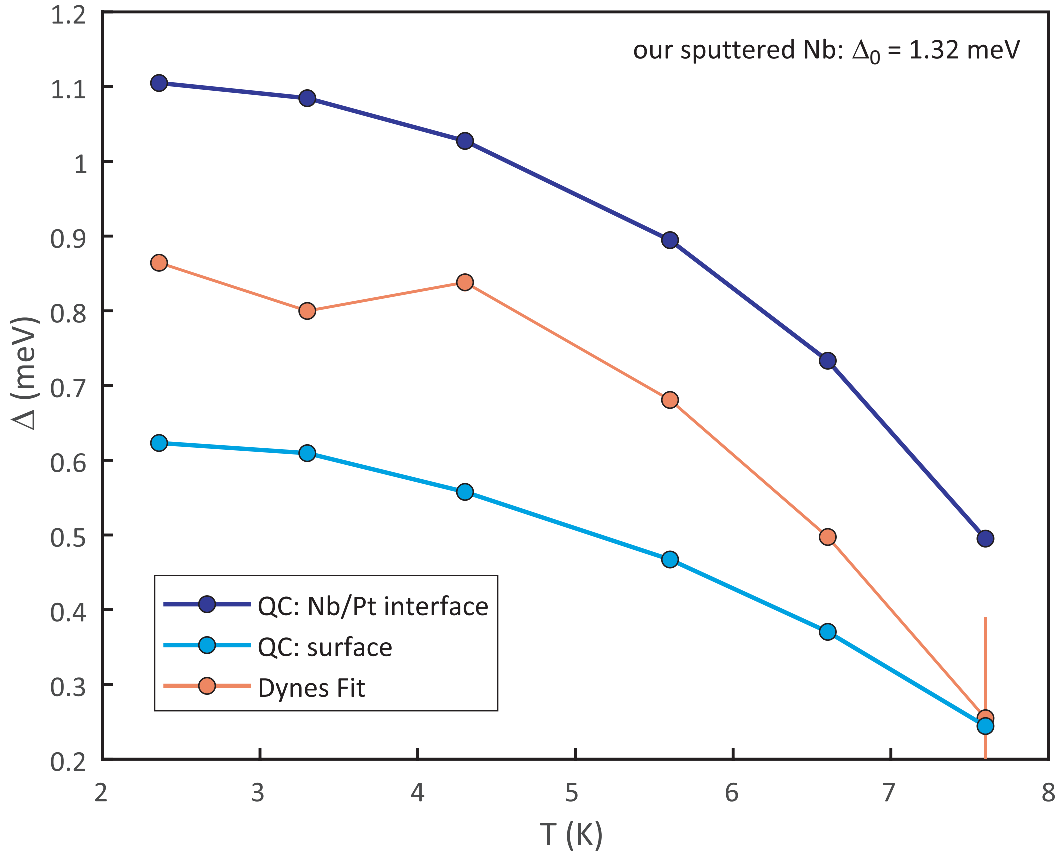

FIG. S2: Temperature dependence of Δ belonging to the curves of Fig. S3 comparing the predictions from the quasiclassical (QC) theory with the Dynes model.

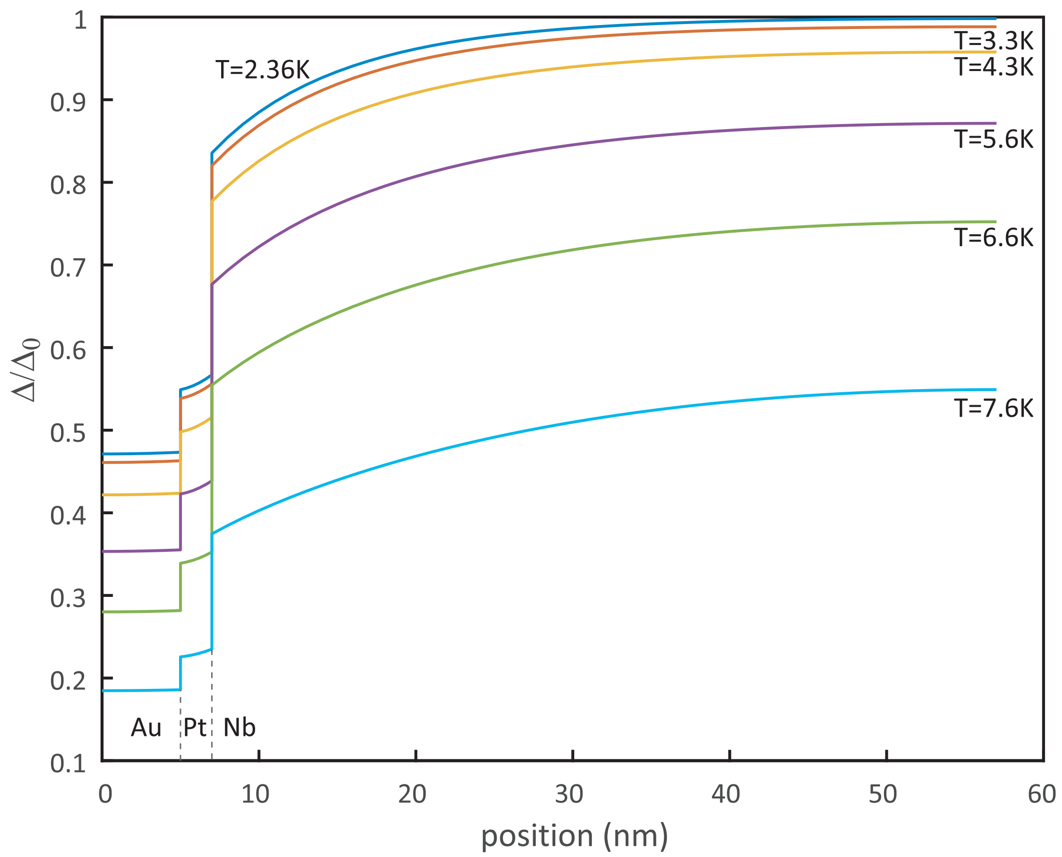

FIG. S3: Spatial dependence of Δ belonging to the (quasiclassical theory) curves of Fig. S3.

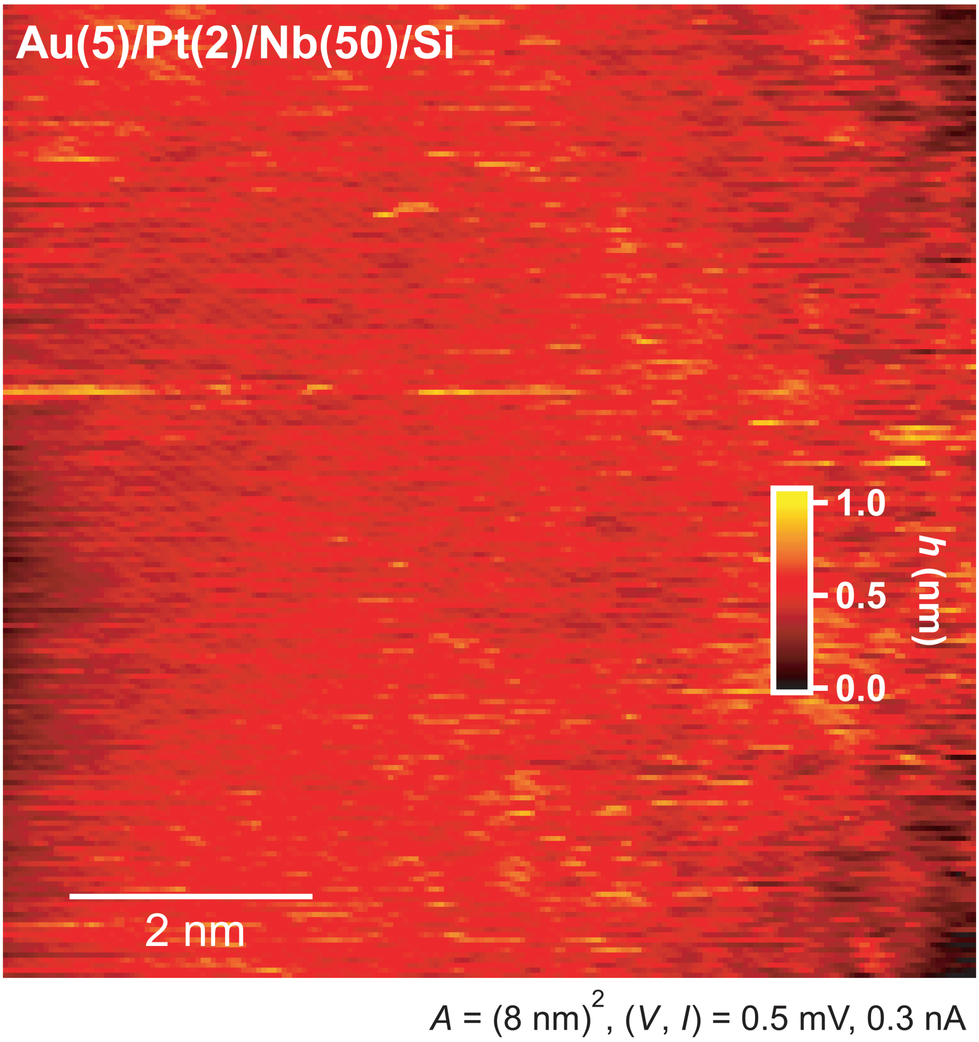

FIG. S4: STM topographic image of the Au(5)/Pt(2)/Nb(50)/Si Sample.

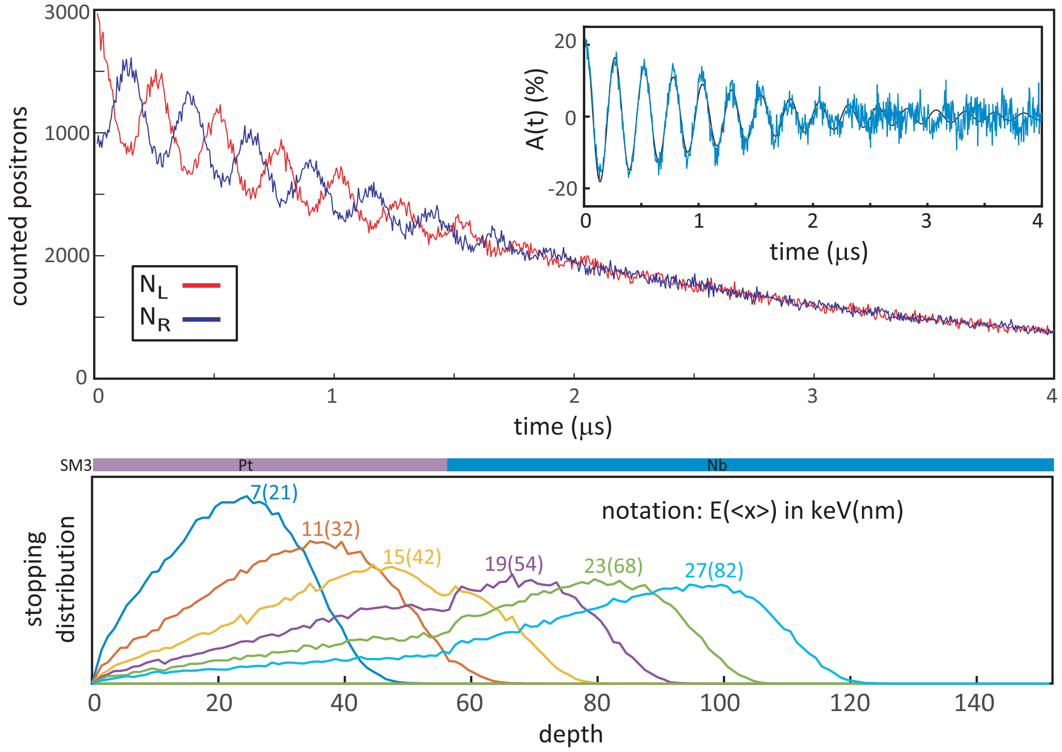

FIG. S5: Top: Example of measured detector histograms $N_{\mathrm{L,R}}(t)$ for the left (L) and right (R) positron detector, taken on the Pt(56nm)/Nb(96nm)/Si (SM3) sample at $T = 2.7$ K, a muon implantation energy of 27 keV, and at an applied field of approximately 300 G. Inset shows the asymmetry $A(t)$ of the signal which carries all the information about the local field distribution. Bottom: muon stopping profile as function of muon implantation energy.

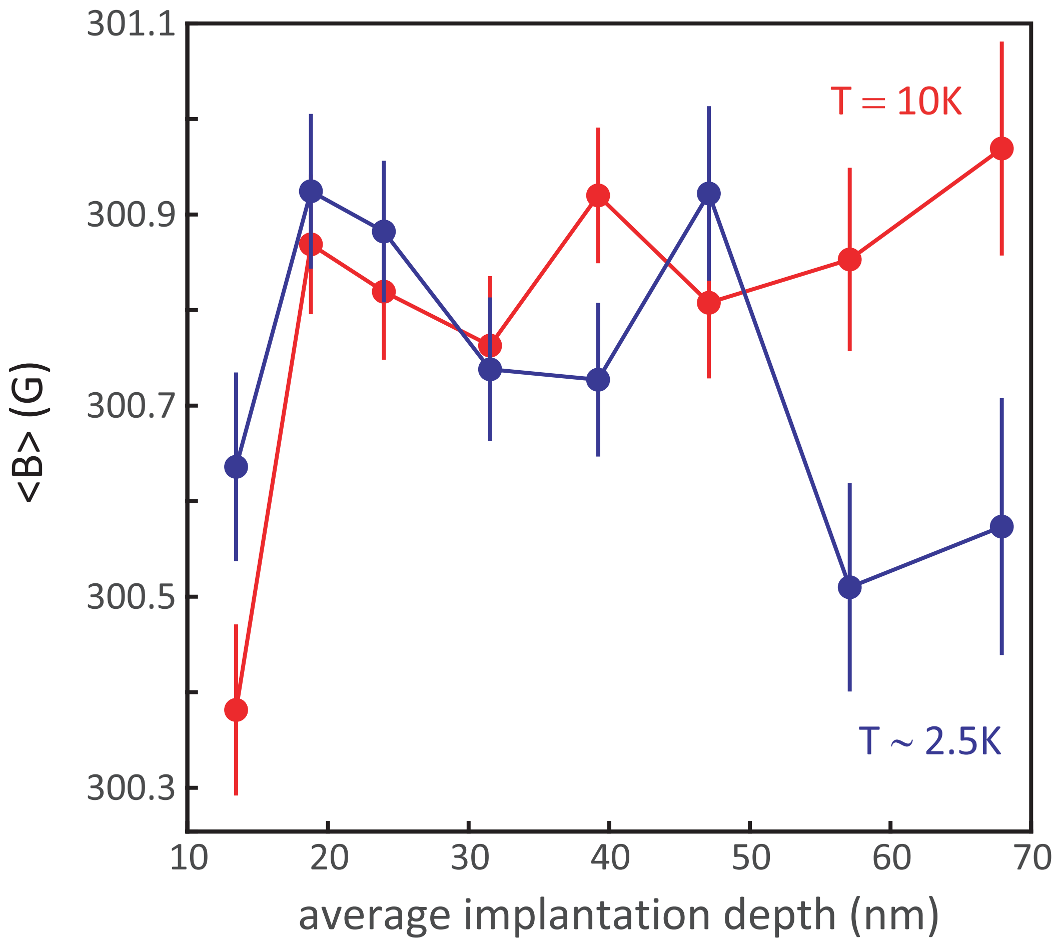

FIG. S6: Low-energy muon-spin rotation data for the average flux density $\langle B \rangle$ as function of average muon implantation depth $\langle x \rangle$ for a Pt(60nm)/Nb(50nm) sample at an applied field of approximately 300 G.

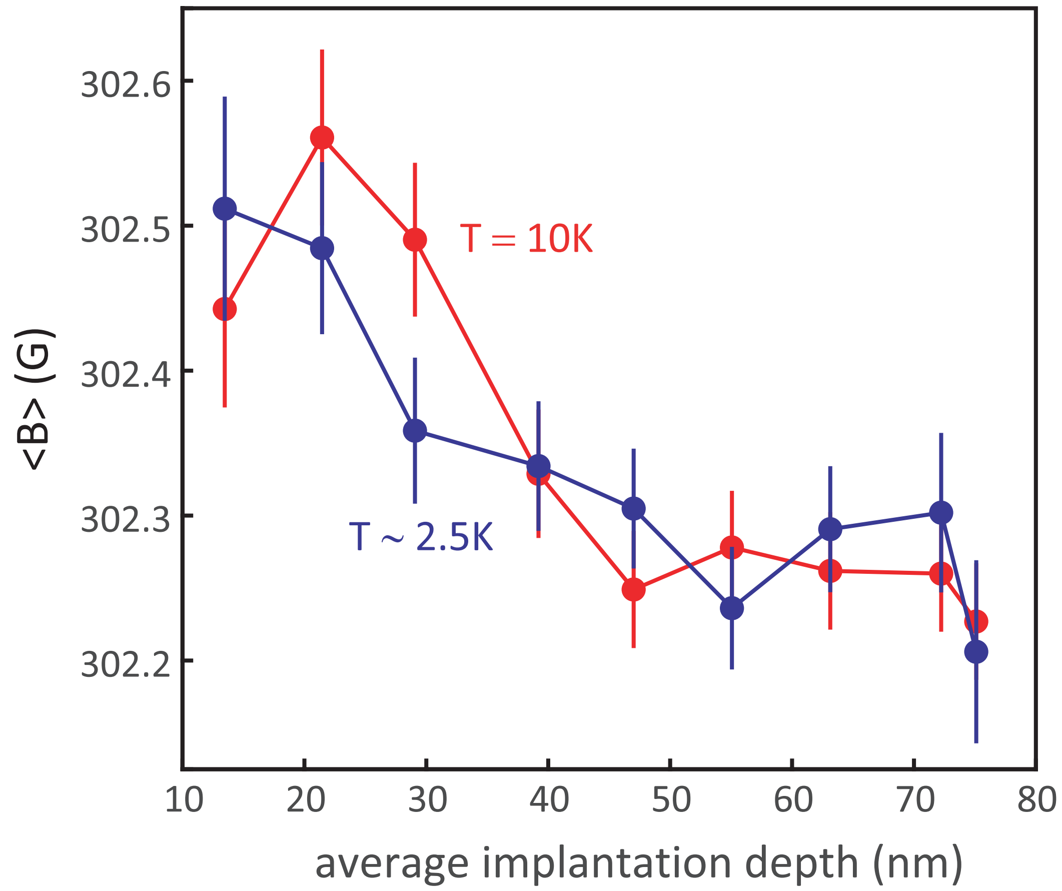

FIG. S7: Low-energy muon-spin rotation data for the average flux density $\langle B\rangle$ as function of average muon implantation depth $\langle x\rangle$ for a Pt(94nm)/Nb(96nm) sample at an applied field of approximately 300 G.

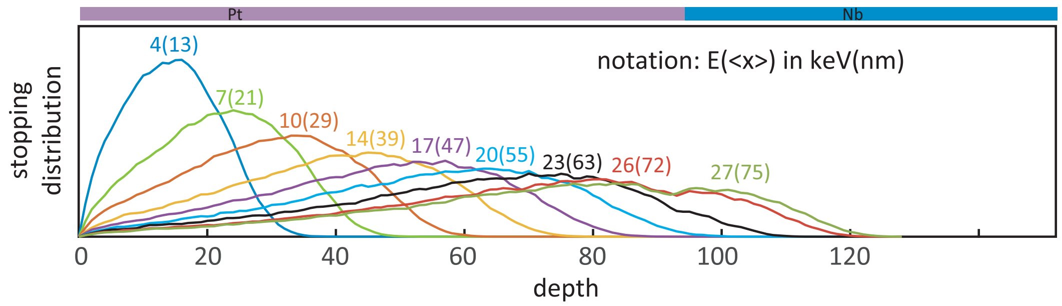

FIG. S8: Muon stopping profile as function of muon implantation energy for out Pt(94nm)/Nb(96nm) sample.

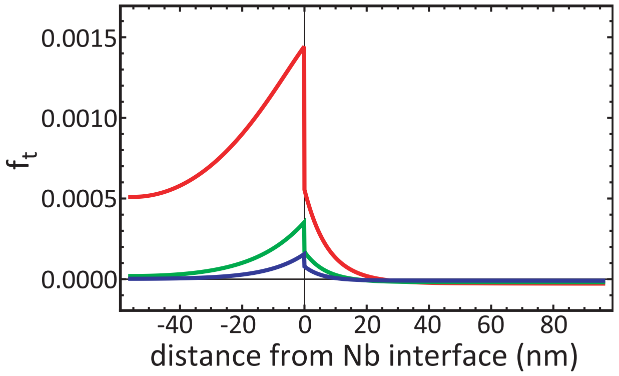

FIG. S9: Triplet contribution to the anomalous Green's function for Pt/Nb system (SM3). The Pt/Nb interface is at $x = 0$. Different curves correspond to different Matsubara frequencies $\omega_0 = \pi T$(red); $\omega_1 = 3\pi T$(green); $\omega_2 = 5\pi T$(blue). $T = 2.5K$.

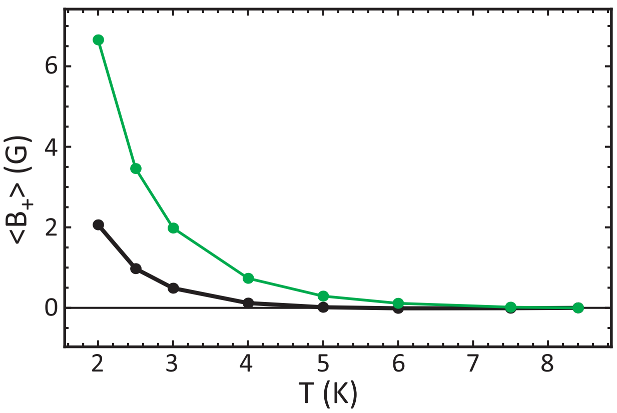

FIG. S10: Convolution of δB with the muon stopping distribution $19(54)keV(nm)$ (green) and with $27(82)keV(nm)$ distribution (black).

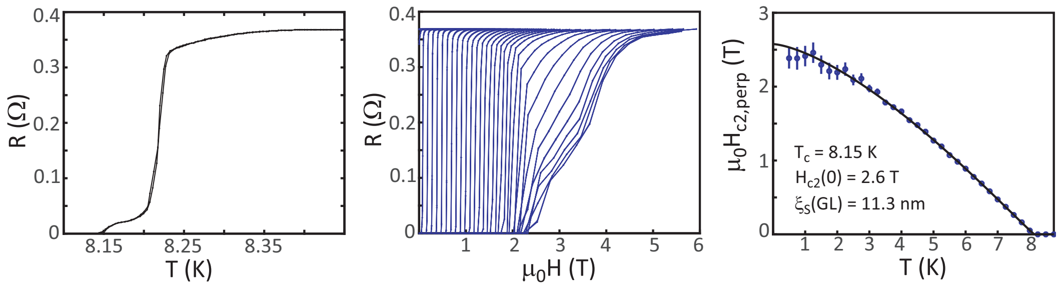

FIG. S11: $R(T)$, $R(H)$ for varying T and its extracted $H_{c2}(T)$ for the Cu/Pt/Nb sample (SM2).

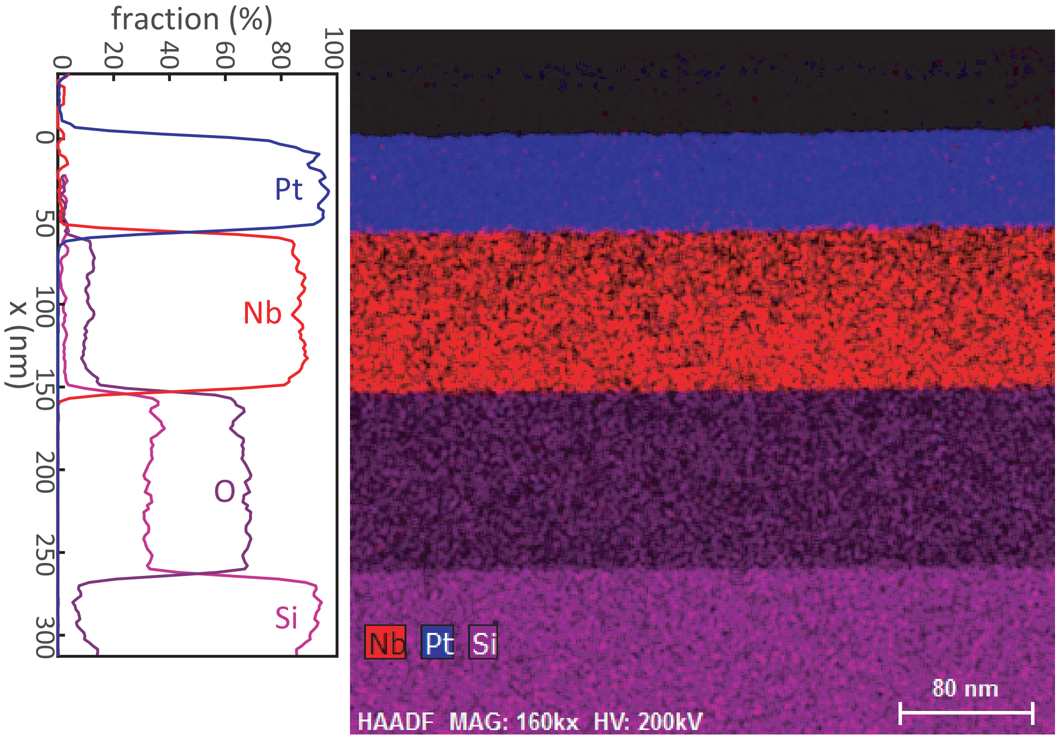

FIG. S12: EDS analysis of the Pt/Nb sample (SM3) showing well defined interfaces and no signs of alloying. The layer between the Nb and Si is a SiO_2 passivation layer.

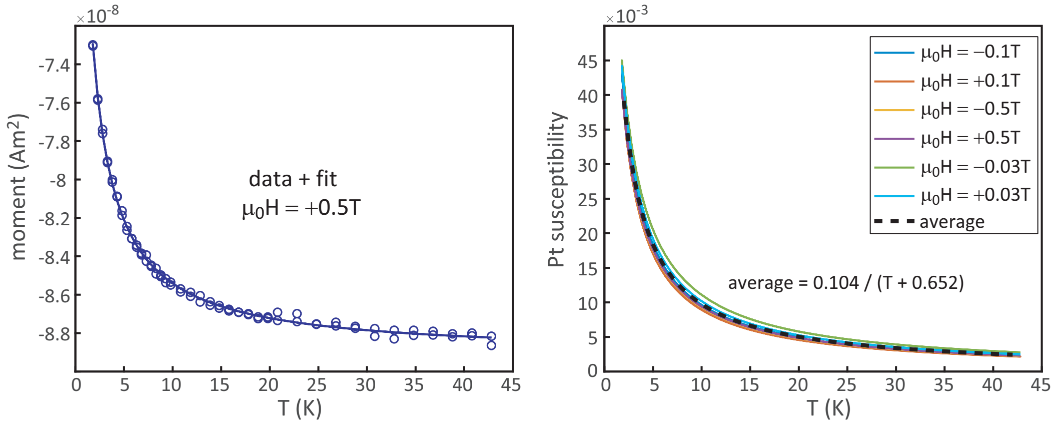

FIG. S13: SQUID magnetometry measurement results to determine the low temperature magnetic susceptibility of a Pt film with a thickness of 40 nm and a surface area of approximately 25 mm^2. Left: obtained low temperature dependence of the Pt moment in a measurement field of 0.5 T. Measurements were taken for both increasing and decreasing temperature and the data (circles) are fit (solid line) using a $A/(T+B)$ dependence. Right: the fit results on all the measurements, taken at measurement fields of ±0.5 T, ±0.1 T and ±0.03 T, and normalized to the respective measurement field to obtain the susceptibility. The average of the fits is shown by the dashed line.

[1] K. D. Usadel. Generalized Diffusion Equation for Superconducting Alloys. *Phys. Rev. Lett.* **25**, 507 (1970).

[2] M. G. Flokstra, R. Stewart, N. Satchell, G. Burnell, H. Luetkens, T. Prokscha, A. Suter, E. Morenzoni, S. Langridge, S. L. Lee. Observation of Anomalous Meissner Screening in Cu/Nb and Cu/Nb/Co Thin Films. *Phys. Rev. Lett.* **120**, 247001 (2018).

[3] Y. V. Nazarov. Novel circuit theory of Andreev reflection. *Superlattices Microstruct.* **25**, 1221-1231 (1999).

[4] O. Narikiyo, H. Fukuyama. Proximity Induced Meissner Effect in Dirty Normal Metals. *J. Phys. Soc. Jpn.* **58**, 4557 (1989).

[5] M. Tinkham. Introduction to Superconductivity 2nd Edition (McGraw-Hill, New York, 1996).

[6] R. C. Dynes, V. Narayanamurti, J. P. Garno. Direct Measurement of Quasiparticle-Lifetime Broadening in a Strong-Coupled Superconductor. *Phys. Rev. Lett.* **41**, 1509 (1978).

[7] T. Prokscha, E. Morenzoni, K. Deiters, F. Foroughi, D. George, R. Kobler, A. Suter, V. Vrankovic. The new μE4 beam at PSI: A hybrid-type large acceptance channel for the generation of a high intensity surface-muon beam. *Nucl. Instr. and Meth. A* **595**, 317-331 (2008).

[8] W. Eckstein. Computer Simulation of Ion-Solid Interactions. (Springer, Berlin, Heidelberg, New York, 1991).

[9] E. Morenzoni, H. Glückler, T. Prokscha, R. Khasanov, H. Luetkens, M. Birke, E. M. Forgan, Ch. Niedermayer, M. Pleines. Implantation studies of keV positive muons in thin metallic layers. *Nucl. Instr. and Meth. B* **192**, 254-266 (2002).

[10] P. Bakule, E. Morenzoni. Generation and applications of slow polarized muons. *Contemporary Physics* **45**, 203 (2004).

[11] F. S. Bergeret, I. V. Tokatly. Manifestation of extrinsic spin Hall effect in superconducting structures: Nondissipative magnetoelectric effects. *Phys. Rev. B* **94**, 180502 (2016).

[12] C. Huang, I. V. Tokatly, F. S. Bergeret. Extrinsic spin-charge coupling in diffusive superconducting systems. *Phys. Rev. B* **98**, 144515 (2018).

[13] P. Virtanen, F. S. Bergeret, I. V. Tokatly. Magnetoelectric effects in superconductors due to spin-orbit scattering: Nonlinear σ-model description. *Phys. Rev. B* **104**, 064515 (2021).

[14] V. M. Edelstein. Triplet superconductivity and magnetoelectric effect near the s-wave-superconductor– normal-metal interface caused by local breaking of mirror symmetry. *Phys. Rev. B* **67**, 020505(R) (2003).

[15] A. G. Mal'shukov, C. S. Chu. Spin Hall effect in a Josephson contact. *Phys. Rev. B* **78**, 104503 (2008).

[16] Z. H. Yang, J. Wang, K. S. Chan. Proximity effect in a superconductor/two-dimensional electron gas junction with Rashba spin–orbit coupling. *Supercond. Sci. Technol.* **22**, 055012 (2009).

[17] C. R. Reeg, D. L. Maslov. Proximity-induced triplet superconductivity in Rashba materials. *Phys. Rev. B* **92**, 134512 (2015).

[18] F. Konschelle, I. V. Tokatly, F. S. Bergeret. Theory of the spin-galvanic effect and the anomalous phase shift ϕ_0 in superconductors and Josephson junctions with intrinsic spin-orbit coupling. *Phys. Rev. B* **92**, 125443 (2015).

[19] I. V. Bobkova, A. M. Bobkov. Quasiclassical theory of magnetoelectric effects in superconducting heterostructures in the presence of spin-orbit coupling. *Phys. Rev. B* **95**, 184518 (2017).

[20] W. Belzig, F. K.Wilhelm, C. Bruder, G. Schön, A. D. Zaikin. Quasiclassical Green's function approach to mesoscopic superconductivity. *Superlattices and Microstructures* **25**, 1251 (1999).

[21] J.W. Serene, D. Rainer. The quasiclassical approach to superfluid ^{3}He. *Phys. Rep.* **101**, 221-311 (1983).
